# Pulsed Laser Deposition of CsPbBr_3_ Films: Impact of the Composition of the Target and Mass Distribution in the Plasma Plume

**DOI:** 10.3390/nano11123210

**Published:** 2021-11-26

**Authors:** Maura Cesaria, Marco Mazzeo, Gianluca Quarta, Muhammad Rizwan Aziz, Concetta Nobile, Sonia Carallo, Maurizio Martino, Lucio Calcagnile, Anna Paola Caricato

**Affiliations:** 1 Department of Mathematics and Physics “Ennio De Giorgi”, University of Salento, 73100 Lecce, Italy; marco.mazzeo@unisalento.it (M.M.); muhammadrizwan.aziz@unisalento.it (M.R.A.); maurizio.martino@unisalento.it (M.M.); annapaola.caricato@unisalento.it (A.P.C.); 2 CNR NANOTEC-Institute of Nanotechnology, c/o Campus Ecotekne, via Monteroni, 73100 Lecce, Italy; concetta.nobile@nanotec.cnr.it (C.N.); sonia.carallo@nanotec.cnr.it (S.C.); 3 National Institute of Nuclear Physics (INFN), 73100 Lecce, Italy; gianluca.quarta@unisalento.it (G.Q.); lucio.calcagnile@unisalento.it (L.C.); 4 CEDAD (Center of Applied Physics, Dating and Diagnostics), University of Salento-Cittadella della Ricerca SS.7, Km 7300, 72100 Brindisi, Italy

**Keywords:** all-inorganic perovskite, pulsed laser deposition, CsPbBr_3_ films, off-stoichiometry aspects

## Abstract

All-inorganic cesium lead bromine (CsPbBr_3_) perovskites have gained a tremendous potential in optoelectronics due to interesting photophysical properties and much better stability than the hybrid counterparts. Although pulsed laser deposition (PLD) is a promising alternative to solvent-based and/or thermal deposition approaches due to its versatility in depositing multi-elemental materials, deep understanding of the implications of both target composition and PLD mechanisms on the properties of CsPbBr_3_ films is still missing. In this paper, we deal with thermally assisted preparation of mechano-chemically synthesized CsPbBr_3_ ablation targets to grow CsPbBr_3_ films by PLD at the fluence 2 J/cm^2^. We study both Cs rich- and stoichiometric PbBr_2_-CsBr mixture-based ablation targets and point out compositional deviations of the associated films resulting from the mass distribution of the PLD-generated plasma plume. Contrary to the conventional meaning that PLD guarantees congruent elemental transfer from the target to the substrate, our study demonstrates cation off-stoichiometry of PLD-grown CsPbBr_3_ films depending on composition and thermal treatment of the ablation target. The implications of the observed enrichment in the heavier element (Pb) and deficiency in the lighter element (Br) of the PLD-grown films are discussed in terms of optical response and with the perspective of providing operative guidelines and future PLD-deposition strategies of inorganic perovskites.

## 1. Introduction

Since the first successful preparation of CsPbX_3_ (X = Cl, Br, I) colloidal nanocrystals by a solution-phase synthesis method in 2015 [1], all-inorganic cesium lead bromide halide perovskites have gathered interest due to higher temperature stability (a melting point higher than 500 °C) and photo-bleaching stability against moisture compared to hybrid organic-inorganic perovskites [2,3,4,5,6]. Due to further advantageous optoelectronic properties, such as wide color tunability, narrow emission bandwidths, high photoluminescence quantum yield and intrinsic tolerance to defects, high mobility and long life time of carriers in CsPbBr_3_ [7,8], all-inorganic halide perovskites are widely investigated as a new generation of materials in future device applications [9,10].

Unlike the prototypical all-inorganic halide perovskite CsPbI_3_ that is unstable under ambient conditions, the excellent photophysical properties of cesium lead bromide (CsPbBr_3_) perovskite [1,11,12] have boosted the research activity towards its applications in numerous fields including photovoltaics [13,14,15,16,17], light-emitting diodes [18,19,20], photodetectors [21,22,23], lasing [24,25], field effect transistors [26] and color-conversion layers [27]. In regard to thermal stability and crystal structure, CsPbBr_3_ perovskite has a yellow orthorhombic phase at room temperature and transforms to a tetragonal phase at 88 °C and to the orange cubic phase at 130 °C [28]. Due to its wide band gap (~2.3 eV), most of the light absorption ability is retained during cubic to orthorhombic phase transition, which implies excellent moisture and thermal tolerance even in 95% humidity conditions and heating up to 100 °C [29,30].

Noteworthy, the performance of the devices based on CsPbBr_3_ was found to be critically dependent on the fabrication technique and involved interface modifications [31].

Nowadays, CsPbBr_3_ nanocrystals and thin films (more suitable than nanocrystals for most integrated optoelectronic device architectures) can be synthesized by various methods, including wet (solution-based) methods and dry vacuum methods [4,32], spin-coating [31,33,34], hot injection [35], solvothermal synthesis [23,36], room-temperature precipitation [31], low-temperature solution-growth [37], Bridgman growth [7], chemical vapor deposition (CVD) [38], vacuum dual source thermal evaporation [10,39,40] and pulsed laser deposition (PLD) [41,42].

Although solution-based bottom-up growth methods leading to CsPbBr_3_ nanocrystals and nanostructured films do not require expensive experimental equipment, the main practical drawbacks are: (i) the usage of hazardous solvents and organometallic reagents as well as eventual capping ligands, (ii) the restricted choice of solvents and their inability to dissolve different reactants effectively, (iii) solvent residuals, (iv) the negative impact of solvents on the morphology and crystallization, (v) the presence of unavoidable pinhole defects, (vi) multi-step processing and (vii) time-consuming synthesis of gram-scale quantities of CsPbBr_3_ precursor powders.

For instance, the sensitivity of the phase diagram of Cs-Br-Pb compounds to any slight deviation from a perfect 1:1 PbBr_2_: CsBr molar ratio leads to either the CsBr-rich composition (i.e., Cs_4_PbBr_6_) or PbBr_2_-rich composition (i.e., CsPb_2_Br_5_) [43]. Additionally, the different solubility of PbBr_2_ and CsBr in aprotic solvents results in the precipitation of CsBr- or PbBr_2_-rich compounds alongside the desired CsPbBr_3_ phase and the low solubility of CsBr salt causes poor element distribution and poor surface coverage [43,44]. Therefore, in practice, solution-based methods yield poor reproducibility, uniformity and control on the desired composition over a large area coverage [27,45].

In order to synthesize CsPbBr_3_ powders to be post-processed to obtain films, mechano-chemical synthesis, consisting in the reaction of halide salts triggered by mechanical energy [46,47], has emerged as an alternative to solution-processing [43,44]. Mechano-chemical synthesis is a completely solvent-free approach yielding CsPbBr_3_ powders [48] to be used in vacuum-deposition techniques that are suitable for manufacturing optoelectronic chips with more precise control on thickness, composition, purity, uniformity and area scalability [37]. Among dry vacuum-assisted methods, vacuum thermal evaporation enables the growth of large-area single and multilayered films with fine control on thickness, uniformity and composition [39]. A main limitation of thermal evaporation for the growth of multication–multihalide materials is the different evaporation rate of the involved source materials; the evaporation rate ratio of CsBr to PbBr_2_ (i.e., 0.5:1–1.2:1 for 25–300 °C) can favor the deposition of an excess of PbBr_2_ depending on the growth temperature [40]. Hence, dual-source thermal evaporation of CsPbBr_3_ films, that exploits the co-evaporation of CsBr and PbBr_2_ source materials from independent crucibles, needs fine calibration of the evaporation rate of the precursors in order to achieve control on the stoichiometric molar ratio of the film [40,49]. On the other hand, sequential single-source vacuum deposition onto a low temperature substrate can result in a two-phase (orthorhombic and cubic) crystal structure requiring post-annealing to drive phase transition, removal of pinholes and formation of high-quality films [39,50]. Additionally, pressure-assisted high-temperature sequential thermal evaporation growth and/or a post-growth annealing treatment can be necessary, due to CsBr precursors poorly reacting properly with PbBr_2_ salt because of their hard diffusion in the growing film [50]. Recently, single source thermal evaporation of CsPbBr_3_ precursor material, prepared by a common solution-based multi-step approach, was reported to yield films consisting of a mixture of CsPbBr_3_, CsPb_2_Br_5_ and Cs_4_PbBr_6_ compounds requiring mild post deposition treatments to obtain full conversion to CsPbBr_3_ [51]. Therefore, post-deposition annealing of the CsPbBr_3_ films deposited by thermal evaporation is a key post-growth step to improve quality and solar cell conversion efficiency [10,40,52]. On the other hand, although single-source vapor deposition is performed using mechano-chemically synthesized halide perovskite powders, different evaporation rates of the multiple components of the compacted target may cause off-stoichiometric transfer.

In this context, the peculiar ability of the PLD vacuum-assisted method to transfer the stoichiometry of multi-elemental materials could represent an interesting possibility of overcoming the limits of the thermal evaporation techniques due to the flexible interplay between the deposition parameters, which allows for a higher degree of control over thickness and phase morphology [53,54,55].

Recently, CsPbBr_3_ thin films were deposited by PLD using targets prepared by pressing a stoichiometric mixture of PbBr_2_ and CsBr powders (molar ratio 1:1) under a pressure of 40 MPa [41]. Although the deposition fluence and target-to-substrate distance are not indicated to achieve a repeatability of the PLD deposition, elemental and morphological characterizations pointed out that a growth temperature of 150 °C is needed to obtain good quality CsPbBr_3_ thin films. It is worth noting that EDS analysis of the structurally optimized film shows a Br-deficient and a slightly Pb-enriched composition, which are not discussed so far.

In addition, CsPbBr_3_ targets to be used in PLD growth-experiments were prepared by single crystal powders, mixed with a 1:2 molar ratio CsBr:PbBr_2_, and grown by an inverse temperature crystallization (ITC) solvent-related method [42]. In this case, PLD-based growth was carried out at a substrate temperature as high as 320 °C and a post-growth annealing at 350 °C for 2 h was applied to improve the film quality [42]. Due to the weight loss of CsPbBr_3_ expected above 250 °C [56], it is not possible to assess the impact on the final film composition of the interplay between the annealing-induced mass loss and the mass distribution related to the characteristics, in terms of distribution of species and angular divergence, of the plasma plume. This point is critical because the mass distribution in the laser plume depends on the elemental composition of the target, ablated species (ions and neutrals), energy of first ionization and number of mass of the species in the plasma plume [53,57,58]. To date, compositional deviations from the required stoichiometry due to mass gradients between organic and inorganic components was reported in the PLD deposition of hybrid perovskite films [59,60].

In the field of all-inorganic perovskites, room temperature PLD was demonstrated to be a successful technique for depositing CsSnI_3_ thin films based on the ablation of a solid target manufactured by mechano-chemical synthesis of CsI and SnI_2_ powders [61]. Despite the evaporation rate being almost different between CsI and SnI_2_ [62], PLD is straightforwardly expected to yield a stoichiometric deposit due to the similar atomic mass of Sn, I and Cs (119, 127 and 133 a.u., respectively). Differently, in the case of CsPbBr_3_, the larger difference in the atomic mass (Pb is much heavier than Cs and Br) may play a role in dictating film composition and homogeneity. The implications of the mass distribution of Cs, Pb and Br in the plume originating in PLD deposition experiments of CsPbBr_3_ was never investigated in the literature, to the best of our knowledge, and represents the main aim of our study. Moreover, in the literature, reaction kinetics and structural/morphological transformations of mechano-chemically synthesized CsPbBr_3_ powders were investigated, mainly in the case of the stoichiometric mixing of the precursor salts, in the absence of thermal treatments and without using the prepared powders to deposit thin films [48]. Therefore, a comprehensive study of performances and issues related to mechano-chemically synthesized pressed CsPbBr_3_ powders used for film growth is not addressed so far.

In this paper, we report on the PLD–based room temperature growth of nearly stoichiometric single-phase cubic CsPbBr_3_ films using targets prepared by thermally-assisted mechano-chemical synthesis. In order to decouple temperature-driven elemental loss and PLD-driven effectiveness of stoichiometry transfer, no substrate heating and post-annealing treatment of the PLD-deposited films was accomplished. In detail, unlike conventional application of the all-solid state mechano-chemical synthesis route [46,48], we combine a non-stoichiometric CsBr:PbBr_2_ molar ratio and temperature treatments (from room temperature to 550 °C) of the target planned to drive the transformation of the spurious phases (Cs_4_PbBr_6_ and CsPb_2_Br_5_) to the desired composition CsPbBr_3_.

A relevant indication of our room temperature PLD experiments is the key role played by the relative mass distribution of the involved elements (Cs, Pb and Br) in the plasma plume that impacts on the stoichiometry of the PLD-deposited films more importantly than a fine control on the target stoichiometry. Indeed, a comprehensive discussion of the results on the basis of the PLD mechanisms demonstrates that off-stoichiometry may occur under different conditions applied to prepare the targets and even under high influence conditions, as a result of a systematic impact of the mass distribution in the plasma plume.

## 2. Experimental Methods

### 2.1. Mechano-Chemical Synthesis of CsPbBr_3_ Pellets

We prepared a series of five targets in the form of solid pellets of compacted powder by the all-solid-state mechanochemical synthesis approach, according to the following processing steps.

Equal amounts (1 g each) of the binary precursors CsBr and PbBr_2_ (corresponding to a PbBr_2_ to CsBr molar ratio of 1:6.7) were mixed in an agate mortar and pestle. The two precursor salts, purchased by Chempur (99.9 grade purity) without further purification, were carefully hand mixed and grounded together for 30 min at room temperature. For comparison, it is reported that mechanochemical treatment lasting 10 min is long enough to drive a chemical reaction in the preparation of CsPbBr_3_ upon manual grinding in an agate mortar [63,64]. Indeed, the observed change in the color of the mixture of cesium and lead bromides from white to orange confirmed the formation of CsPbBr_3_.

Afterwards the powder mixture was moved under a hydraulic press in a boron nitride target holder where the application of 6-ton pressure through hydraulic press turned the powder mixture into a compressed solid pellet (hereafter termed Target (RT), where RT stands for “room temperature”). In order to assess the impact of temperature-assisted synthesis on the target for PLD experiments, further pellets were prepared by mimicing the synthesis route applied to prepare the target (RT) apart from mixing and grounding lasting 15 min rather than 30 min at room temperature. Then the pressed pellets were transferred to an oven for thermal treatment, without using a sealed fused silica ampule. In this respect, heating temperature was set at the values T_th_ = 300, 400, 500, 550 °C for 2 h and then the oven was switched off and targets were allowed to cool down to room temperature for 2 to 3 h depending on the annealing temperature. The described procedure resulted in the pellets hereafter termed Target (300 °C), Target (400 °C), Target (500 °C) and Target (550 °C), respectively, depending on the applied heating temperature.

Following the above detailed synthesis protocol, all pellets were stored in plastic box and kept within a vacuum ball until their usage in PLD experiments. Noteworthy, all targets were observed to have orange color and, in particular, Target (400 °C) exhibited dark brownish corners, Target (500 °C) had a dark brown-yellow to slightly orange color and Target (550 °C) was orange-colored.

In addition to mixing PbBr_2_ and CsBr with an excess of CsBr, a further target was prepared by mechano-chemical synthesis starting from molar ratio 1:1 of the precursor powders and applying a heating temperature of 550 °C for 2 h for comparison with Target (550 °C). Such a target will be termed Target (550 °C, 1:1) by detailing the changed mixing ratio with respect to Target (550 °C).

### 2.2. Pulsed Laser Deposition Experiments

**Figure 1:**
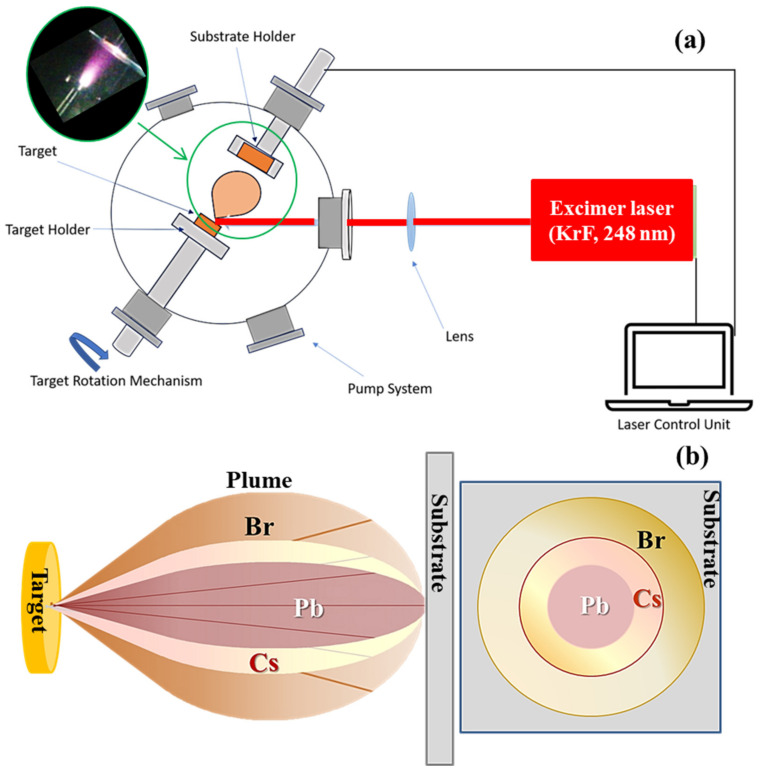
(**a**) Schematic diagram of a PLD experimental set-up. (**b**) Schematics of the angular and mass distribution of light and heavy species (Br, Cs and Pb) in the plasma plume generated under laser ablation of a CsPbBr_3_ target. The left and right panels show a side view and the cross-sectional view of the trace over the substrate, respectively, of the plasma plume formed by ablation of a CsPbBr3 target.

The schematic diagram of a PLD deposition set-up using an excimer laser is shown in Figure 1a. It includes: a stainless-steel vacuum chamber, an excimer (KrF) laser source, laser beam delivery lenses, a combined rotative-turbo pump system to evacuate the chamber down to high-vacuum conditions and a substrate holder placed in front of the target holder. The working principle of the PLD approach can be summarized as follows [65]: a focused pulsed laser beam hitting the source material to be deposited (termed target) may induce, under proper fluence (i.e., energy delivered per pulse and per unit surface) conditions, the generation of a highly forwardly-peaked plasma plume including the species ejected from the target due to the ablation mechanism. The hyperthermal energy distribution of such species enables their transfer from the target to the substrate surface where they deposit and contribute to the film growth.

In our experiments, a focused KrF pulsed laser beam (Lambda Physics laser equipment), with operating wavelength λ_KrF_ = 248 nm and pulse duration τ_p_ = 20 ns, was incident at 45° with respect to the surface of the mechanochemically synthesized targets mounted on a rotating target holder. Such a rotation allows the avoidance of the drilling and formation of craters on the irradiated area.

Before starting the film deposition, the stainless-steel chamber was evacuated down to a background pressure as low as ∼10^−5^ Pa and the target surface was cleaned by 1000 laser pulses fired at 10 Hz in the presence of a shutter masking the substrate from contaminations. The ablated species were collected on a silica substrate, pre-cleaned in acetone and ethanol for 10 min at 60 °C each, placed at the distance of d_T-S_ = 4 cm from the target. PLD depositions of interest in this study were performed at room temperature, fluence of 2 J/cm^2^ and 5 J/cm^2^, frequency of 5 Hz and by firing 2000 laser pulses.

In regard to the nomenclature adopted hereafter, the PLD-grown films will be referred to as Film (300 °C) and Film (550 °C) if deposited by laser ablation of Target (300 °C) and Target (550 °C), respectively. When turning to Target (550 °C, 1:1), the associated films, deposited by PLD under different fluence conditions (2 J/cm^2^ and 5 J/cm^2^), will be named Film (2 J/cm^2^, 1:1) and Film (5 J/cm^2^, 1:1).

Table 1 lists the samples (mechanochemically synthesized targets and PLD-deposited films) under discussion in this study together with the associated preparation parameters and measured composition.

### 2.3. Characterization Analyses

#### 2.3.1. X-ray Diffraction (XRD) Analysis

X-ray Diffraction XRD spectra (Rigatu Company, Tokio, Japan) were acquired using a Cu Kα X-ray source of wavelength 1.54056 Å. The instrument worked at the voltage of 40 KV and operating current of 40 mA. The X-ray pattern of the specimen of interest was acquired over the angular range 2θ = 10–40°.

#### 2.3.2. Scanning Electron Microscopy (SEM), Energy-Dispersive Spectroscopy (EDS) and Atomic Force Microscopy (AFM) Analyses

Elemental composition and morphological investigations of the samples under consideration in the present work were conducted, respectively, on the SEM microscopes: a JEOL-JSM-6480LV (JEOL Ltd., Tokyo, Japan), with an integrated XEDS system for a microanalysis purpose, and a FE-SEM ZEISS Merlin (Carl ZEISS, Oberkochen, Germany, GmbH), operating with an accelerating voltage of 10 kV and short acquisition times of a few seconds, for sample damage minimization. The percentage uncertainty in the chemical composition estimated by EDS was found to be of < 2% on the relative weight of each atomic species investigated

Atomic Force Microscopy (AFM) topography images were measured by a Park XE-70 Instruments microscope (Park Systems, Suwon, Korea) operating under non-contact mode at room temperature in air environment. The scan size was set as 10 × 10 μm^2^. Average roughness R_a_ is provided to characterize the roughness characteristics of the PLD-deposited films.

#### 2.3.3. Rutherford Backscattering (RBS), X-ray Fluorescence (XRF) Analyses

Rutherford backscattering spectrometry (RBS) was implemented to determine the stoichiometry and thickness of the deposited CsPbBr_3_ film. RBS measurements were carried out at the in-vacuum IBA beamline at CEDAD (Centre of Applied Physics, Dating and Diagnostics) University of Salento [66].

The sample were irradiated with 2.0 MeV He^2+^ particles. Backscattered particles were detected at an angle of 170° by using a PIPS (Passivated Implanted Planar Silicon), detector by Canberra, Montigny-Le-Bretonneux, France [67]. Data acquisition was carried out by using the Canberra Genie Software (GENIE™ 2000, Basic Spectroscopy Software by Canberra Industries, Meriden, CT, USA) and experimental data were fitted by the SIMNRA code (version 7.0, Matej Mayer, Max Plank Institute for Plasma Physics, Garching, Germany) [68].

X-Ray fluorescence (XRF) analyses were carried out at CEDAD by using a portable EDXRF (Energy Dispersive XRF) system supplied by Amptek (AMPTEK, Bedford, MA, USA). X-Rays were produced by using a Mini-X Coolidge X-Ray tube equipped with 0.75 μm Ag target and a 127 μm thick Be end window. Fluorescence X-ray radiation was detected at CEDAD by using an Amptek X-123 spectrometers (AMPTEK, Bedford, MA, USA) based on a Peltier cooled SDD (Silicon Drift Detector), 25 mm^2^ detector with an energy resolution of 135 eV at 5.9 keV. During each measurement the tube voltage and current were set at 35 kV and 5 μA, respectively. The measurement time was set to 600 s. 

#### 2.3.4. Ellipsometry, Photoluminescence and Absorbance Measurements

Real and imaginary parts of refractive index (n, k) of the perovskite samples under consideration in this study were carried out by ellipsometric measurements performed by means of a J. A. Wollam M-2000XI ellipsometer (J. A. Wollam, AL, USA). The shift angles were fitted by using general oscillators (genosc function).

Photoluminescence (PL) spectrum was acquired by a solid-state continuous-wave (CW) laser delivering 100 mW at 405 nm with a diameter spot of 2 mm. The emission signal was collected in backscattering configuration by an optical fiber connected to a compact CCD (Oceanoptics) delivering a PL signal to the whole UV-Vis-NIR spectral window.

Absorbance spectra were measured by a PerkinElmer Lambda 900 UV spectrophotometer (PerkinElmer Company, Waltham, MA, USA) over the wavelength range from 400 to 750 nm and with a wavelength resolution of 3 nm.

## 3. Results and Discussion

### 3.1. Preliminary Remarks

Reaction kinetics and morphological transformations of mechano-chemically synthesized CsPbBr_3_ powders were investigated in the literature in the case of room temperature mixing of a stoichiometric quantity of the precursor salts [48]. On comparing dry mechano-chemical ball-milling with hand-grinding of PbBr_2_ and CsBr precursor powders, it was reported that the former approach may allow a better control on the reaction kinetics and formation of intermediate phases for short processing times [48]. The ball-milled samples demonstrated morphological changes as well as a worsening of the photoluminescence performances for ball milling times longer than 5 min, because of defect states induced by the process [48]. On this basis and accounting for the documented complete reaction into CsPbBr_3_ occurring in less than 5 min for 1:1 (PbBr_2_:CsBr) molar ratio, we applied hand grinding, which is a simpler procedure requiring no additional accessories, and exploited thermal annealing to drive phase transformations.

To better explain the reasoning underlying our experiments we observe that, in practice, the phase diagram of a CsBr-PbBr_2_ mixture favors the formation of three ternary compositions, namely CsPbBr_3_ and the spurious phases Cs_4_PbBr_6_ and CsPb_2_Br_5_ in the presence of a slight deviation of the 1:1 molar ratio [43,69]. On the other hand, thermal annealing at T > 220 °C can drive the reaction [56,70].

CsPb_2_Br_5_
→ CsPbBr_3_ + PbBr_2_ (1)

In this case, the reaction product PbBr_2_ can be removed by a reversible reaction or by the following one.

3 PbBr_2_ + Cs_4_PbBr_6_ → 4 CsPbBr_3_ (2)

Hence, as the formation energy of Cs_4_PbBr_6_ is lower than the Cs_2_PbBr_5_ one [46,48], the removal of the spurious ternary phases is expected to be favored by the combination of two factors/processes. First, a starting Cs-rich composition of the PbBr_2_-CsBr mixture drives the dominant formation of Cs_4_PbBr_6_ with respect to CsPb_2_Br_5_. Second, thermal annealing at a proper temperature exploits the above reactions to drive the formation of the desired CsPbBr_3_ single phase. Therefore, in our experiments we mixed equal weight amount of PbBr_2_ and CsBr powders, corresponding to a CsBr-rich mixture, and progressively increased the annealing temperature of the mixture to drive consumption of the spurious phases according to the above reported reactions. Noteworthy, since Cs-rich composition of the mixed precursor powders was deliberately used in our experiments, unreacted impurities would occur even if mechano-chemical ball-milling is used instead of hand-grinding. Further, the presence of unreacted phases is not a limit because both Cs-rich ternary phase and PbBr_2_ residuals are exploited to thermally drive the transformation to the desired phase.

Our approach is expected to enable a proper understanding of the stoichiometry transfer process from the target to the substrate in the presence of mass differences [58,71]. Additionally, elemental evaporation driven by thermal annealing in an oven can progressively reduce the CsBr-excess and favor the formation of CsPbBr_3_ phase. Moreover, as the transformation from the hexagonal Cs-rich Cs-Pb-Br phase to cubic CsPbBr_3_, at low temperature and in Pb-lean conditions, may be accompanied by the formation of structural defects, it could be preferable to thermally drive the transformation Cs_4_PbBr_6_ → CsPbBr_3_ in the ablation target rather than in the deposited film.

Therefore, hereafter we will both present a comprehensive characterization of the mechanochemically synthesized targets under consideration in this study and discuss the stoichiometry issue of PLD accounting for the differences between properly chosen targets and the associated PLD-grown films. In particular, the importance of the mass ratios on the issue of the stoichiometric transfer will be discussed in detail. The growth by PLD of multicomponent materials including heavy and light elements may be critical due to the dependence of the cation ratio on the process parameters (fluence, background pressure, substrate temperature and so on) [53,58,71]. To date, the distribution of ions and neutral species composing the plume is ruled by a cosine power-law (i.e., ~cos^n^ θ) and a superposition of cosine and cosine power-law (i.e., ~A cosθ + B cos^n^ θ), respectively [58]. For increasing value of the n-power, the species concentrate more along the central part of the plume. As the n-power varies proportionally to the sublimation energy and mass number, the mass distribution of ions is expected to be Pb, Cs and Br on going from the expansion axis to the periphery of the plume (Figure 1b).

As our experiment plasma plume expands in vacuum and the substrate is kept at room temperature, any impact of gas-plume collisions and gas-induced confinement of the plume as well as re-evaporation from the substrate can be ruled out. Additionally, the target-to-substrate distance is set (d_TS_ = 4 cm), meaning that, at a given fluence, the distance travelled by the ablated species is not an experimental variable. Therefore, the stoichiometric composition of the deposited films may only depend on the interplay between target composition and/or effects of the ablation mechanisms and plume-mediated transfer dynamics of heavy and light species.

### 3.2. Impact of Thermal Heating on the Preparation of the Targets

**Figure 2:**
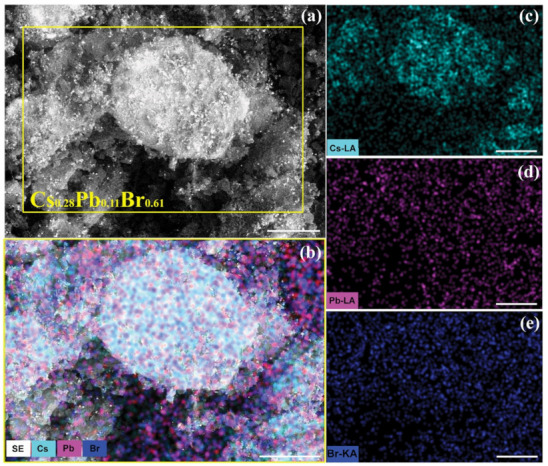
Morphological and elemental compositional characterization of Target (RT). (**a**) SEM micrograph image of a representative region of Target (RT). (**b**) Overlap of the SEM image pointed out by a yellow rectangle in panel (**a**) and the corresponding Cs, Pb and Br EDS maps reported in panels (**c**) for Cs, (**d**) for Pb, and (**e**) for Br elements. (Scale bars (**a**–**e**): 200 μm).

Figure 2 reports on the morphological and elemental SEM and EDS characterizations of Target (RT). SEM micrograph in Figure 2a presents a heterogeneous morphology of the target featured by (a few hundreds of μms of) large structures. Such microstructures have irregular shapes and make the target surface porous and wrinkled. Moreover, it can be noticed that the surface roughness is increased by the presence of small agglomerates of particulates, with sizes from a few hundreds of nanometers to a few microns, that are randomly and widely distributed along the entire surface of the large microstructures. XEDS-based elemental mapping, revealing chemical composition, coupled to SEM analysis (Figure 2b) points out the spatial distribution of the Cs, Pb and Br elements throughout the morphological features of the Target (RT).

By considering the high-energy lines for each element (Br(K), Cs(L) and (Pb(L) as shown in Figure 2c–e), an uneven distribution of the mapped elements can be clearly observed. While the Cs distribution clearly adapts to the morphology, Pb deficiency can be observed over the micrometer-sized features, where larger thickness doesn’t correspond to a more intense signal. It looks that Pb concurs to the signal stemming from the fine-grain agglomerates and particulates.

A chemical composition Cs_0.28_Pb_0.11_Br_0.61_ (Table 1) can be inferred by the EDS analysis. Such a composition is consistent with Pb-deficiency and Cs-excess, as expected in the case of the Cs-rich mixture of the precursor powders. The measured deviation from the stoichiometric composition (i.e., Cs_0.20_Pb_0.20_Br_0.60_) of CsPbBr_3_ can be accounted for the occurrence of multiple phases, as it will be discussed hereafter based on XRD analysis.

**Figure 3:**
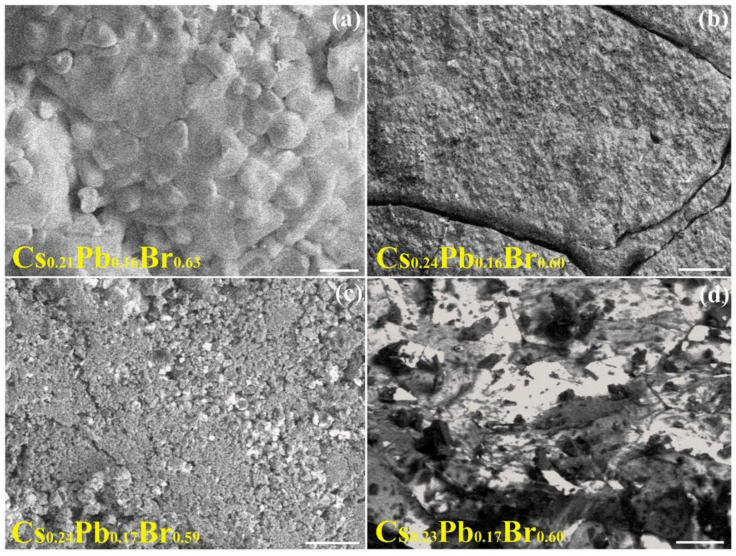
SEM images of the mechano-chemically prepared targets for increasing annealing temperature: (**a**) Target (300 °C), (**b**) Target (400 °C), (**c**) Target (500 °C) and (**d**) Target (550 °C). (Scale bars (**a**–**d**): 100 μm).

Figure 3 shows how the increasing annealing temperature affects the morphology of the mechano-chemically prepared targets. Target (300 °C) (Figure 3a) resembles the morphology of Target (RT) with larger hundreds of micron-sized structures covered by smaller rounded grains with size of a few microns. Such smaller grains can be the result of diffusion and thermal-promoted agglomeration/melting of the sub-μm-sized particulates exhibited by Target (RT). Increasing the annealing temperature from 300 °C to 400 °C (Figure 3b) causes the disappearance of the large grains and enhances agglomeration/coalescence of the smaller (tens of micron large) particulates.

The resulting topography consists of large domains, encapsulating grains randomly distributed onto a mostly homogeneous and continuous surface. The presence of both larger (one or two tens of microns) and narrower (a few microns) extended cracks can be observed in Target (400 °C) (Figure 3b). Such cracks likely originate from thermal stress effects. The Target (400 °C) surface results densely covered by sub-μm-sized granular agglomerates too. A composition Cs_0.21_Pb_0.16_Br_0.63_ for Target (300 °C) and Cs_0.24_Pb_0.16_Br_0.60_ for Target (400 °C) can be extracted by the analysis of their respective compositional EDS data (Table 1). Indeed, the compositional data in Table 1 associated with the samples from Target (300 °C) to Target (550 °C) indicate Cs/Pb and Cs/Br ratios showing, apart from slight fluctuations, a composition closer to the desired one of CsPbBr_3_ than Target (RT). Hence, accordingly to the structural evolution that XRD analysis reported hereafter confirms, increasing the annealing temperature is expected to drive the transformation towards the desired CsPbBr_3_ phase. Consistently, on the basis of the characteristic morphology of the Cs-Pb-Br phases, Target (400 °C) could be composed of CsPbBr_3_ rounded grains surrounded by fine grains with less developed morphology.

Further thermal treatment of (Target (500 °C) in Figure 3c) leads to the formation of non-uniformly distributed, differently sized, coarse grains, and to a reduction of the density and extension of the cracks, favored by the porous character of the sample. A slight change in the heating temperature turning from Target (500 °C) (Figure 3c) to Target (550 °C) (Figure 3d) corresponds, conversely, to a remarkable change in their corresponding morphology. The formation of irregularly-shaped compact large grains and the absence of smaller particulates covering the surface as well as the presence of local surface wrinkles in Target (550 °C), as compared to Target (500 °C), could most likely be explained as a consequence of melting-induced coalescence/agglomeration effects. Elemental EDS analyses of the data related to Target (500 °C) and Target (550 °C) are compatible with a composition of Cs_0.24_Pb_0.17_Br_0.59_ and Cs_0.23_Pb_0.17_Br_0.60_, respectively (Table 1).

Noteworthy, the above reported composition was calculated by means of the normalized atomic percentage (at%) resulting from EDS measurements and accounts for the fact that the content of the involved elements sum to 100%. The raw data associated with Target (400 °C) and Target (550 °C) let one observe a mass loss of all the involved elements, that can be ascribed to a mass loss effect of CsPbBr_3_ observed at a temperature higher than 350 °C [72,73]. This phenomenon would be expected due to high temperature thermal annealing performed in a conventional oven without keeping the target within a sealed fused silica ampule. Hence, while increasing annealing temperature would promote the consumption of the spurious phases, according to the above indicated reactions, elemental loss is also favored under our experimental conditions. However, since the composition of the targets annealed at a temperature higher than 400 °C is dominated by the CsPbBr_3_ phase (see XRD spectra), high-temperature promoted decomposition of CsPbBr_3_ in practice results in losses of the weight of the target without critically affecting its composition in terms of CsPbBr_3_

**Figure 4:**
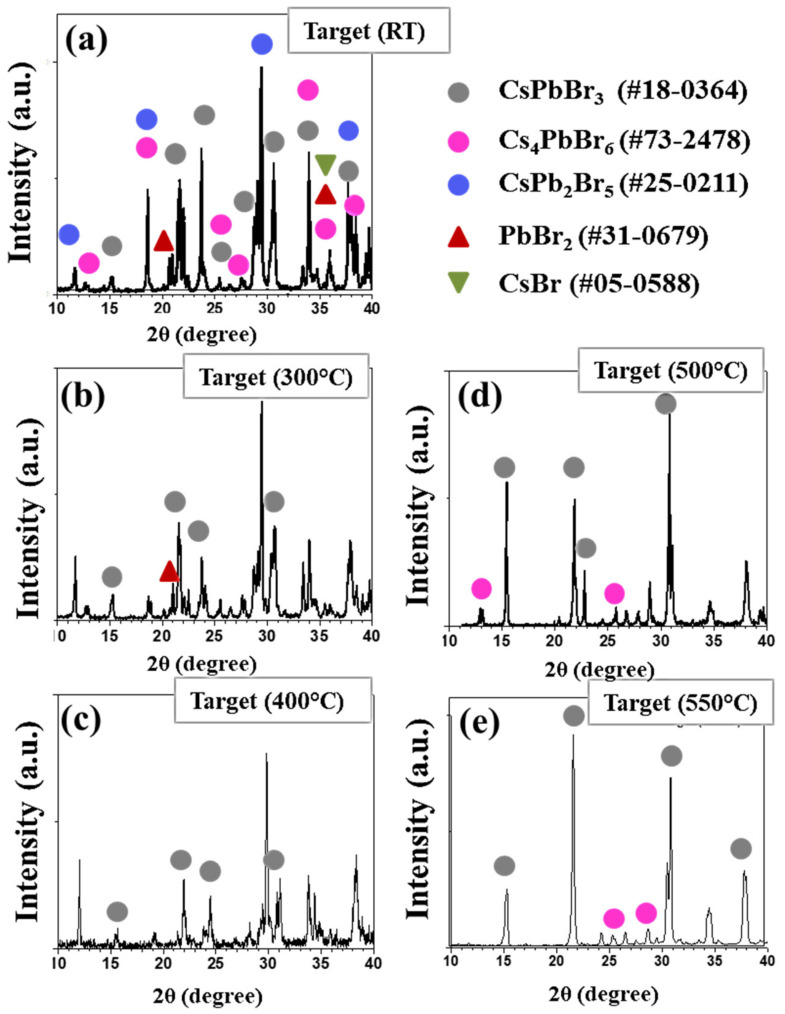
XRD spectra of the mechano-chemically synthesized targets under different heating treatments, that is (**a**) room temperature, (**b**) 300 °C, (**c**) 400 °C, (**d**) 500 °C and (**e**) 550 °C.

Crystallinity and phase information were studied by XRD analysis. Peaks occurring in a not-overlapping region of the XRD pattern, including minor intensity reflections, are a convenient guide for the identification of the occurring Cs-Pb-Br compounds. The XRD diffractogram of Target (RT) (Figure 4a) presents peaks corresponding to all three ternary compositions, that is, the stoichiometric orthorhombic CsPbBr_3_ (pdf card #18-0364), rhombohedral Cs-rich Cs_4_PbBr_6_ (pdf card #73-2478) and tetragonal Pb-rich CsPb_2_Br_5_ (pdf card #25-0211) [37,74]. The peaks located at 2θ = 15.18, 21.55, 30.69, 37.80 deg correspond to the characteristic peaks of CsPbBr_3_ associated with (100), (110), (200) and (211) crystal lattice planes (pdf card #18-0364) of the cubic phase, respectively [75]. The peak split at nearly 15.16 and 30.28 degrees may indicate the formation of the orthorhombic phase, meaning the two-phase CsPbBr_3_ coexistence [40]. On the other hand, the peaks at 2θ = 11.7, 29.38 degrees correspond to the (0 0 2) and (2 1 3) lattice planes of the CsPb_2_Br_5_ (pdf card #25-0211), and the XRD features at 2θ = 12.7 degrees can be associated with the Cs-rich Cs_4_PbBr_6_ phase.

Previous literature discussing the preparation by the mechano-chemical route reported the presence of the intermediate products CsPb_2_Br_5_ and Cs_4_PbBr_6_ at the initial stages of the synthesis. Our XRD data not only specify the peaks corresponding to the phases of the ternary Cs-Pb-Br system but also show some peaks indicating the presence of residual unreacted PbBr_2_ (pdf card #31-0679) and CsBr (pdf card #05-05282). The presence of Cs_4_PbBr_6_ and residual CsBr can be ascribed to the Cs-rich composition of the starting PbBr_2_-CsBr mixture as well as to the formation energy being lower for Cs_4_PbBr_6_ than for CsPb_2_Br_5_. In general, the occurrence of all ternary and binary phases can be accounted for hand grinding as responsible for inhomogeneous mixing.

Turning from Target (RT) to Target (300 °C) (Figure 4b), decreased contributions from CsPb_2_Br_5_, Cs_4_PbBr_2_ and PbBr_2_ compounds can be explained based on the temperature driven (T > 220 °C) reaction given by Formula (1) combined with the reaction given by Formula (2) [76].

The targets heated at a temperature higher than 300 °C show residual presence of spurious phases and a progressive evolution towards the CsPbBr_3_ composition (Figure 4b). For annealing temperatures higher than 400 °C (Figure 4c–e), the intensity of the peaks at 2θ = 15.18, 21.55, 30.69 and 37.80 degrees increases and predominates, meaning that the pellets evolve towards CsPbBr_3_ as the main phase and the occurrence of a few dominant orientations, such as (100) and (200). It is worth noticing that, whereas a residual Cs_4_PbBr_2_ phase can still be observed in the XRD spectra of Target (500 °C) (Figure 4d) and Target (550 °C) (Figure 4e), mechano-chemical synthesis conducted at 550 °C lets one obtain an almost single phase CsPbBr_3_ target, despite the excess of CsBr in the starting powder mixture.

### 3.3. PLD-Deposited CsPbBr_3_ Films: Impact of the Thermal Treatment of the Target

The next step of our investigation was to consider PLD-grown films deposited by using Target (300 °C) and Target (550 °C) as representative targets. Target (300 °C) exhibits multi-phase composition and XRD diffractogram with similar features as compared to Target (RT) and Target (400 °C). As a result of the thermal treatment, Target (550 °C) presents the more relevant improvement in term of a single CsPbBr_3_ phase.

**Figure 5:**
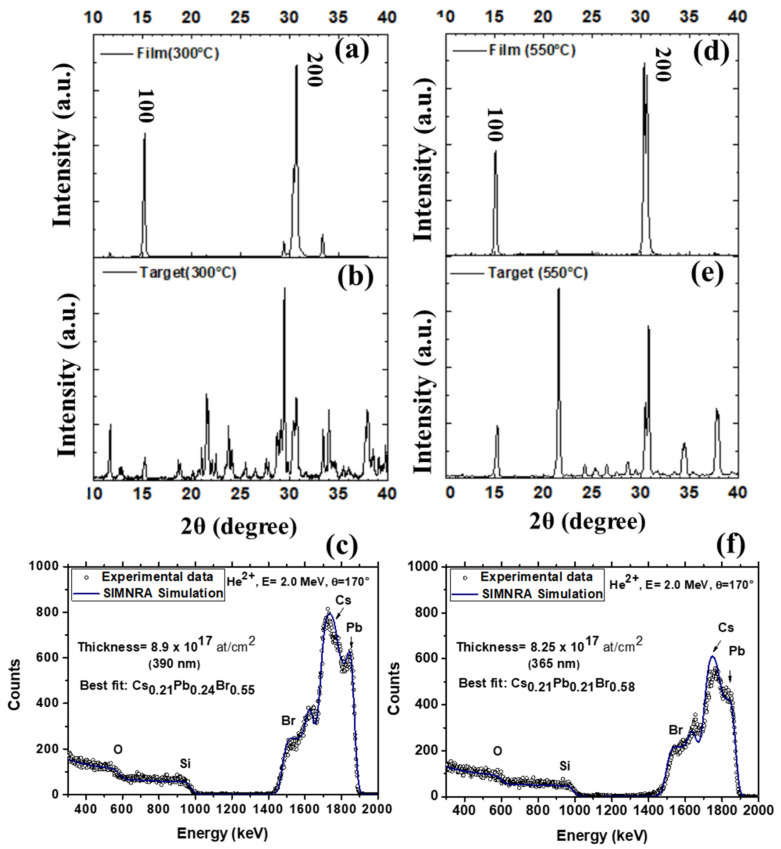
XRD and compositional RBS characterization of (**a**–**c**) Film (300 °C) and associated Target (300 °C) and (**d**–**f**) Film (550 °C) and associated Target (550 °C).

Figure 5 reports on the XRD spectra of Film (300 °C) (Figure 5a) and Film (550 °C) (Figure 5d) compared to the XRD spectra of the associated ablation targets, that is, Target (300 °C) (Figure 5b) and Target (550 °C) (Figure 5e). Despite the discussed differences between Target (300 °C) and Target (550 °C) in terms of phase components and composition, a relevant issue of our experiments is that both Film (300 °C) and Film (550 °C) are CsPbBr_3_ and single-phased with the common textures, (100) and (200). Indeed, minor satellite peaks due to residual Cs_4_PbBr_3_, with negligible intensity, occur in the XRD spectra and RBS measurements demonstrated composition Cs_0.21_Pb_0.24_Br_0.55_ for Film (300 °C) (Figure 5c) and Cs_0.21_Pb_0.21_Br_0.58_ for Film (550 °C) (Figure 5f) (Table 1). The thickness estimation by RBS was 390 nm for Film (300 °C) and 365 nm for Film (550 °C).

Off-stoichiometry between target and substrate can be evaluated by means of the composition ratio r(Pb/Br) defined by the following formula:

$$r\left(\mathrm{Pb}/\mathrm{Br}\right)=\frac{{\left(\mathrm{Pb}/\mathrm{Br}\right)}_{\mathrm{film}}-{\left(\mathrm{Pb}/\mathrm{Br}\right)}_{\mathrm{target}}}{{\left(\mathrm{Pb}/\mathrm{Br}\right)}_{\mathrm{target}}}$$

where (Pb/Br) refers to the ratio between the content of Pb and Br. It resulted in r(Pb/Br) = 0.69 for Film (300 °C) and r(Pb/Br) = 0.34 for Film (550 °C). These estimations indicate a larger off-stoichiometry between target and substrate for Film (300 °C) than for Film (550 °C), in accordance with the temperature-driven modification of the target stoichiometry discussed by EDS. Indeed, as the composition of Film (300 °C) and Film (550 °C) is closer to the desired composition Cs_0.20_ Pb_0.20_ Br_0.60_ than the composition of Target (300 °C) and Target (550 °C), the composition ratio quantifies the compositional deviation between film and target in terms of the CsPbBr_3_ phase. Target (300 °C) and Target (550 °C) being multi-phased and nearly CsPbBr_3_-phased, respectively, accounts for the larger value of r(Pb/Br) in the case of Target (300 °C) versus Film (300 °C).

One aspect highlighted by our experiments that needs deeper insight is the presence of a Pb surface layer, a few nanometers thick, pointed out by RBS-fitting spectra of both Film (300 °C) and Film (550 °C). To date, an excess of Pb combined with an increase of the Br content, corresponding to surface terminated by PbBr_2_, was reported in very small CsPbBr_3_ nanocrystals synthesized by solvent-base methods in the presence of surface stabilizing organics [73]. Due to the relevant difference between our systems and dispersed nanocrystals, we considered an alternative mechanism to address the superficial segregation of Pb. The interplay between cohesive energy per atom (2.03 eV/atom for Pb, 4.32 eV/atom for Cs and 1.22 eV/atom for Br) and heat of fusion (4.77 kJ/mol for Pb, 67.74 kJ/mol for Cs and 15.44 kJ/mol for Br) of the involved elements can be invoked [77]. Under laser-induced heating, as Pb can change its phase from solid to melt more easily than Cs and Br, Pb-Pb bonds may be favored by the relatively low cohesion energy of Pb. Noteworthy, surface rich in the light element was reported in the case of BiVO_4_ films grown by PLD and ascribed to preferential ablation of Bi allowed by its high vapor pressure playing a role under conditions favoring evaporation with respect to effective ablation (such as close to ablation threshold fluence) [78]. In our experiments fluence being set at the value F = 2J/cm^2^ which is well above the ablation threshold, Pb preferential ablation can be ruled out. Hence, Pb-enrichment of the film surface could originate from the formation of Pb-Pb surface bonds in the presence of pending bonds, probably favored by surface Pb- enrichment resulting from loss of the Br light element, as will be discussed hereafter.

### 3.4. Impact of the PLD Mechanisms on the Formation of CsPbBr_3_ Films

The above results point out differences between the composition of the target and the final composition of the film that indicates the role of other processes in dictating the dynamics of transfer of the ablation species through the plasma plume. A relationship between off-stoichiometry of the film and the atomic mass ratios of the elements in the target is widely documented in the literature. In the presence of heavy and light elements, compositional deviations are reported depending on the combined effect of several deposition parameters [58,79]. In particular, it is accepted that laser ablation of materials with composition combining heavy and light species may result in as-grown deposits deficient with respect to the light components due to off-stoichiometry of the forefront plasma plume [58,79]. In the case of multi-elemental targets, ions and neutrals ablation products of each element are involved in intra-plume interactions and travel with mass-dependent velocity. Hence, their arrival time on the substrate and mean free path may be different and losses of the light components of the plume expanding in vacuum may be favored for increasing both mass ratios and target-to-substrate distance, which affect the angular spread of the plume expansion and its trace on the collector surface.

In our experiments, the target-to substrate distance is comparable to the value given in studies reporting compositional deviations depending on the relevant mass ratios [58,71]. The comparison between the composition Cs_0.21_Pb_0.24_Br_0.55_ of Film (300 °C) (Table 1) and the composition Cs_0.21_Pb_0.16_Br_0.63_ of the associated ablation target (i.e., Target (300 °C)) (Table 1) clearly indicates Pb-enrichment and Br-loss on turning from the target to the film. Based on the multi-component nature of the Cs-Pb-Br compounds, including heavy and light elements (Pb, Cs and Br have atomic mass 207, 133 and 80 a.u., respectively), the above experimental result can be rationalized by scattering effects of the light plume-species according to the following picture.

Laser ablation decomposes the irradiated multicomponent target into a distribution of energetic ions and neutrals that fly towards the substrate collector surface where they rearrange, driven by high surface mobility. As the elemental transfer from the target to the substrate is mediated by the plasma plume, it is worth mentioning the mechanism of mass distribution in the plume. As the distribution of ions and neutral species composing the plume is ruled by a cosine power-law and a superposition of cosine and cosine power-law, respectively [57], for the same value of the n-power occurring in both laws, neutrals are distributed more toward the lateral side of the plume and ions are distributed more closely to the plume axis. For increasing value of the n-power, the species concentrate more along the central part of the plume. As the n-power varies proportionally to the sublimation energy and mass number, the multi-elemental composition of the target material needs to be taken into account.

On the basis of the energy of first ionization (that is, 3.9 eV for Cs, 7.4 eV for Pb and 11.8 eV for Br) as compared to the photon energy of the laser beam (i.e., 5 eV corresponding to 248 nm), Cs is expected to have the higher fraction of ions compared to neutrals. As the first ionization energy of Pb is the highest one among the elements under consideration, the contribution from neutrals will dominate the contribution from ions. The lighter element Br will be in between Cs and Pb. Therefore, based on the dependence of the n-power value on the mass number, the mass distribution of ions is expected to be Pb, Cs and Br on going from the expansion axis to the periphery of the plume, as reported in Figure 1b. Cs and Br being lighter elements than Pb, they can be thermalized faster and scattered into a wide angular range resulting in broadening of the angular distribution of the plasma plume [80,81]; they can also diffuse over longer distances than Pb, once deposited on the substrate. Undoubtedly, it is expected that Br can suffer from more effective scattering effects than Cs, due to both its lighter mass and unconfined expansion of the side region of the plasma plume in vacuum. As a rough estimation, an inverse relationship between the atomic mass and scattering angle of an element was reported in the literature [58]. Noteworthy, diffusion of the lighter species to larger angles promoted by intra-plume scattering events keeps an enhancement of the heavier species along the plume-axis direction.

**Figure 6:**
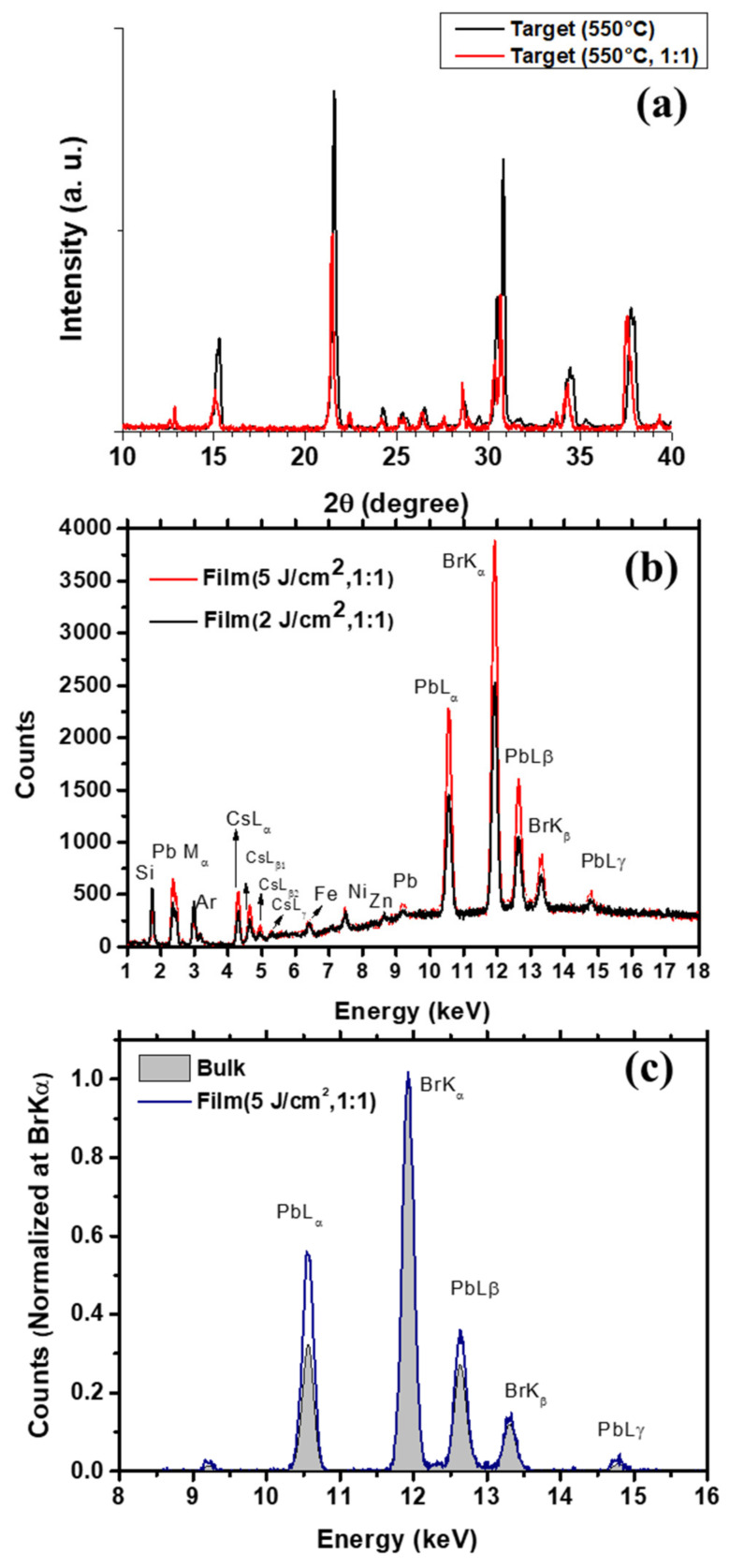
(**a**) XRD spectrum of Target (500 °C, 1:1) compared to Target (500 °C). XRF analysis. (**b**) Comparison between (**a**) Film (2 J/cm^2^, 1:1) and Film (5 J/cm^2^, 1:1), and (**c**) Film (5 J/cm^2^, 1:1) and bulk CsPbBr_3_ material.

As a further step of our investigation, we performed further PLD experiments by setting the conventionally used CsPb:PbBr_2_ molar ratio (i.e., 1:1) to prepare the mechano-chemically synthesized target referred to as Target (550 °C, 1:1). On comparing the XRD spectra of Target (550 °C, 1:1) and Target (550 °C) in Figure 6a, the former target exhibits less refined peaks slightly shifted and traces of the Cs_4_PbBr_6_ phase, that is expected due its formation energy being lower than the one of CsPbBr_3_.

The stoichiometry of the associated PLD-deposited film was characterized by RBS. As a result of RBS fitting, the composition of Film (2 J/cm^2^,1:1) was measured to be Cs_0.22_ Pb_0.22_Br_0.56_ closely to the trace of the plume-axis and Cs_0.24_ Pb_0.22_Br_0.54_ far apart 1 cm from the plume-axis (corresponding to an angle of 14° with respect to the plume axis). Such findings confirm Br-deficiency and Pb-enrichment. Additionally, given the slight decrease in the Br percentage while moving far from the plasma plume-axis, it can be argued that Br species suffer from large angle scattering effects that work against their collection on the substrate. On comparing the composition of Film (2 J/cm^2^,1:1) with Film (550 °C) (Table 1), it can be observed that using a target with a molar ratio CsBr:PbBr_2_ of 1:1 doesn’t provide a stochiometric film. This finding indicates that, for growing CsPbBr_3_ films by PLD, thermal treatment of a CsBr-rich mechano-chemically synthesized target performs better than using the conventional 1:1 composition of the CsBr-PbBr_2_ mixed target.

Further, on keeping into account the key role of fluence in controlling the cation stoichiometry for values well above the ablation threshold [82], further PLD depositions were performed under increasing fluence conditions.

Figure 6b compares XRF spectra of Film (2 J/cm^2^, 1:1) and Film (5 J/cm^2^, 1:1), deposited for CsBr:PbBr_2_ molar ratio of the target set at 1:1 and fluence increased from 2 J/cm^2^ to 5 J/cm^2^. All the spectra were acquired with the same total number of excitation photons as controlled by keeping constant the current in the X-ray tube and the measurement time. The increased intensity of the characteristic fluorescence lines corresponding to Cs, Pb and Br for increasing fluence in Figure 6b is then consistent with the fluence-dependent increased deposition rate. The measured Fe, Ni and Zn signals are related to the experimental set-up. This is also the reason why their intensity is the same between the spectra acquired for the two films. The comparison between the spectra associated with CsPbBr_3_ (labeled as bulk) and Film (5 J/cm^2^, 1:1) in Figure 6c, after subtracting the background and normalizing at the Br K_α_ line, allows a comparison of the relative concentrations of the different elements. By this approach, an enrichment can be clearly claimed for Cs and Pb.

The effect of fluence on film stoichiometry was also checked by EDS analysis that provided the composition Cs_0.20_Pb_0.23_Br_0.57_ in the case of Film (2 J/cm^2^, 1:1) (Table 1) and Cs_0.22_Pb_0.23_Br_0.55_ for Film (5 J/cm^2^, 1:1) (Table 1), thus confirming that increased fluence does not solve for the discussed off-stoichiometry and the critical role of different relative masses.

### 3.5. Morphological and Spectral Investigation of PLD-Grown CsPbBr_3_ Films

Once the issue of the composition of PLD-grown is addressed depending on the mechanism of the PLD techniques, it is worth observing that film morphology is also critical in perspective of photonic applications [30]. On this basis, hereafter we present the results of our investigation of the morphology of the PLD-deposited films and discuss it as related to the PLD technique.

**Figure 7:**
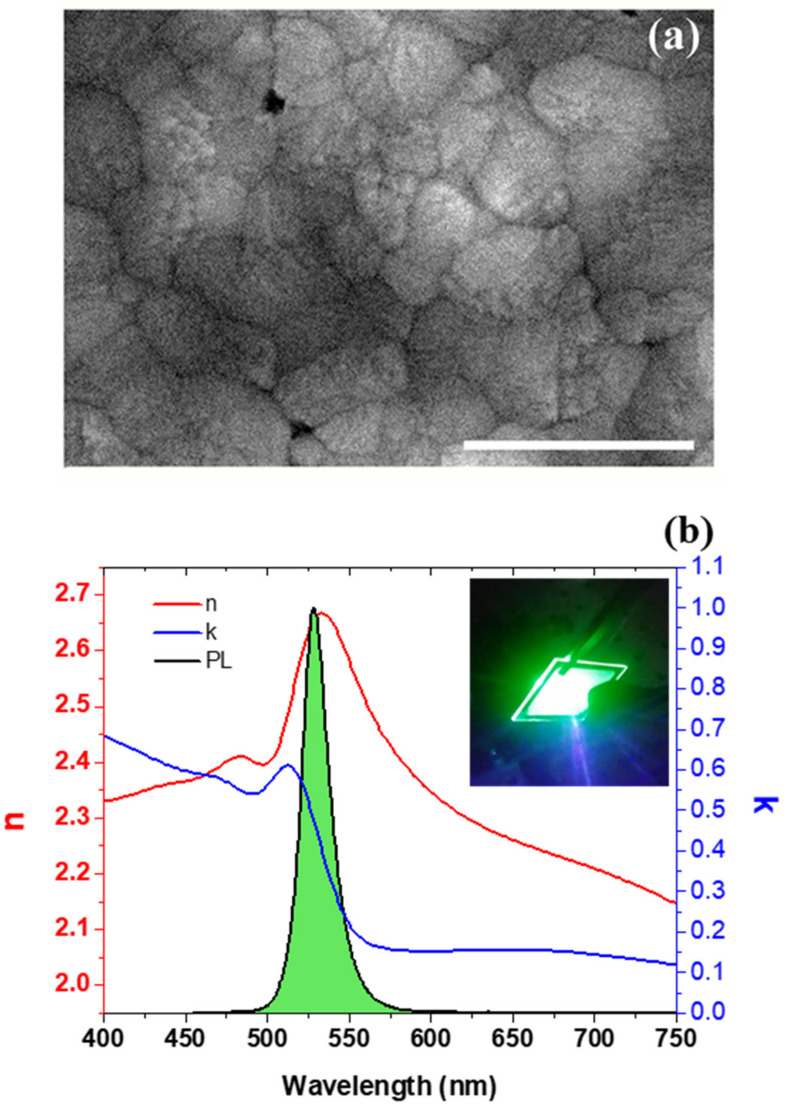
Morphological and optical analysis of Film (550 °C). (**a**) SEM image of a magnified region of the Film (550 °C) (Scale bar: 500 nm). (**b**) Refractive index (red curve), extinction coefficient (blue curve) and PL spectrum (green peaked feature) carried out by exciting the sample by a diode laser operating at a wavelength of 405 nm. The inset of panel (**b**) reports a picture of the emitting sample.

The morphological characterization and spectral response of Film (550 °C) are reported in Figure 7, respectively, as parts (a) and (b). A high-magnified SEM image in Figure 7a shows the occurrence of a granular-like arrangement of the film. Grains, with different shape and size, the latter ranging from tens of nanometers to a few hundreds of nanometers, show some slight surface roughness, and appear spatially arranged to form mostly a compact and homogeneous film without any significant void. Clearly observable coalescence effects can be ascribed to nucleation seeds connection favored by increasing number of laser pulses with the progression of the PLD deposition.

Figure 7b reports on the PL spectrum of Film (550 °C), which accounts for the emission response, and both real and imaginary parts of the refractive index (n, k) resulting from ellipsometric measurements for information mainly about the absorption properties through the extinction coefficient k. The inset shows a picture of the light-emitting film.

By comparing the extinction coefficient k with PL emission, we can conclude that excitonic peak may play an important role, due to the high energy binding between the opposite carrier (electron-hole), despite the high dielectric constant, which tends to reduce the coulombic energy bond. Refractive index n is in line with the value reported in literature. Noteworthy, by transmittance measurements it can be observed that the scattering due to large light-diffusing grains shadows the Gaussian shape of the transition excitonic peak, which is almost evident by extracting optical data by means of k ellipsometer measurements, where a typical sharper line is observed.

**Figure 8:**
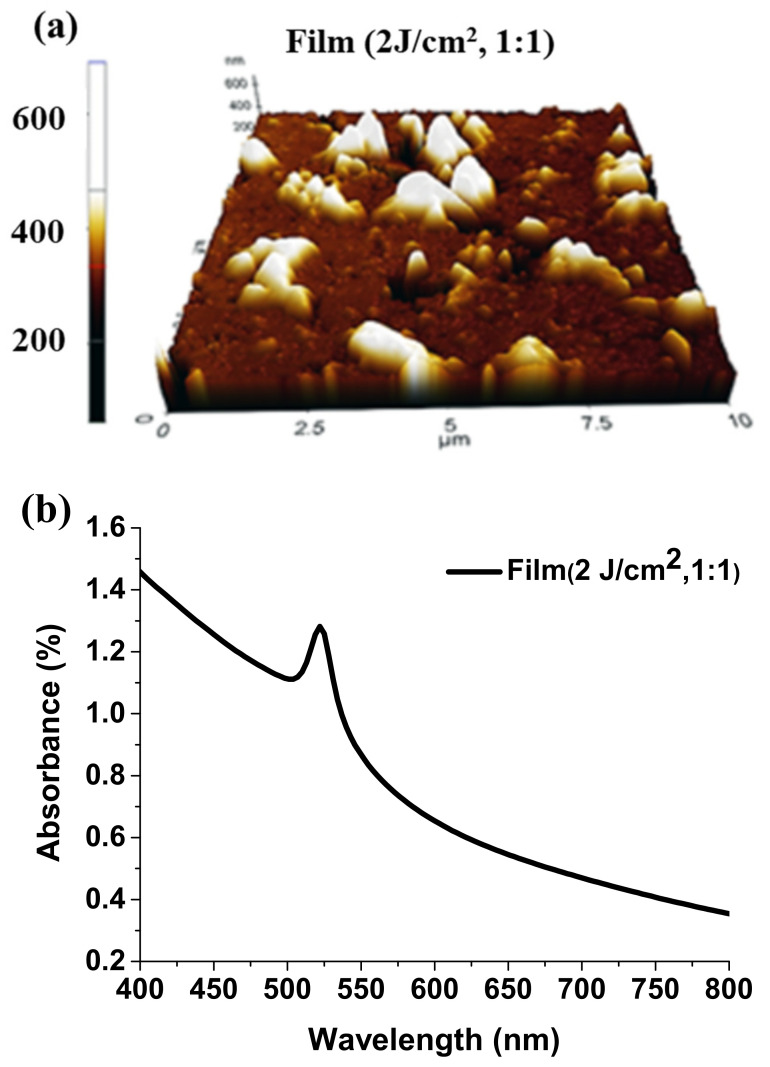
Topographical and optical information on Film (2 J/cm^2^, 1:1). (**a**) 3D AFM topography measured over a scan area of 10 × 10 μm^2^. (**b**) Absorbance spectrum.

For the sake of completeness, morphological and spectral characterizations of Film (2 J/cm^2^, 1:1) are also reported in Figure 8. AFM topography acquired over a 10 × 10 μm^2^ scan area (Figure 8a) shows granular-like film architecture composed by two differently sized perovskite grains, similarly to Film (500 °C). It can be observed that the occurrence of a two-fold structure consisting of a sub-micron sized background is dispersed in between groupings of 3D columnar islands. AFM-based roughness analysis lets one evaluate the average roughness as R_a_~43 nm. Film thickness was estimated to be 530 nm.

The absorbance spectrum of Film (2 J/cm^2^, 1:1) in Figure 8b exhibits a noticeable peak around 520 nm, that can be attributed to the exciton close to the optical bandgap of the CsPbBr_3_ compound [44]. The spectra are a convolution of free-carrier absorption, exciton transition (namely the peak around 520 nm) and hyperbolic wavelength-dependence due to large light-diffusing grains that produce scattering. Unstable and inhomogeneous green emission was observed in this case, which can be ascribed to the differences in composition between Film (550 °C) and Film (2 J/cm^2^, 1:1) (Table 1).

Definitively, in PLD experiments the uniformity of the deposit may be affected by several factors, among which critical factors are both the mass composition of the plume and plume being inherently forwardly peaked. These factors impact on both film thickness and composition. Therefore, inhomogeneities in thickness and composition may be practical consequences of PLD experiments. If an offset between the substrate axis and the plume axis is set and the substrate is rotated while depositing, then uniformity in thickness over a few centimeter squares can be recovered. The occurrence of uneven masses, as it occurs in our experiments, causes a compositional inhomogeneity that, in general and in principle, can be removed by enriching the target in the light element. Therefore, addressing the occurrence of eventual inhomogeneities is the first step and proposing strategies to overcome this issue is the second step. In our study we point out this aspect in the case of CsPbBr_3_ PLD-deposited films and suggest a strategy to limit serious Br-losses by preparing targets with stoichiometry different from the commonly used one. Multiple measurements and/or measurements performed in different points of the sample (for instance, the composition of Film (2 J/cm^2^,1:1) is Cs_0.22_ Pb_0.22_ Br_0.56_ closely to the trace of the plume-axis and Cs_0.24_ Pb_0.22_ Br_0.54_ far apart 1 cm from the plume-axis as related to Pb-enrichment and Br-losses) are useful to disclose undesired inhomogeneities and their impact on the film properties.

## 4. Conclusions

PLD–based room temperature growth of CsPbBr_3_ films has been discussed by using mechano-chemically synthesized targets with different compositions (Cs-rich and stoichiometric) and subjected to thermally assisted treatments. Once temperature-driven elemental loss has been discounted by room temperature PLD deposition, the critical aspects of the off-stoichiometry in depositing CsPbBr_3_ films have been discussed comprehensively. A relevant finding of our room temperature PLD experiments has been that obtaining pure monoclinic CsPbBr_3_ films by PLD is not necessarily related to the target composition, meaning that properly designed thermal treatment of an off-stoichiometric target may allow one to obtain a film with the desired composition. Indeed, a main result of our experiments is that the film composition is sensitive to the target composition because we had to use Cs-rich target to deposit nearly stoichiometric films. Noteworthy, our discussion indicates that the annealing-driven reactions (see Formulas (1) and (2)) are critical to favor phase transformations and consumption of the spurious phases. As these reactions require specific phases, the initial precursor ratio does directly propagate into the target composition through being beneficial or not in driving the needed transformation reactions at a given processing temperature. For instance, we checked several PbBr_2_: CsBr molar ratios and thermal annealing protocols and observed successful PLD-deposition of CsPbBr_3_ in the case of a relevant excess of CsBr in the starting mixture, as reported herein.

A few relevant operative guidelines resulting from our experiments are the following:

For fluence F = 2 J/cm^2^, PLD-deposition of CsPbBr_3_ films is affected by compositional deviations between target and substrate, due to the simultaneous presence of heavy and light species. In addition to differences in the spatial location within the plume due to relevant mass ratio, intra-plume scattering events also play a role, resulting in film enriched in the heavy element and deficiency in the light element. In particular, our findings indicate that, (i) a conventional 1:1 molar ratio target yields a slightly off-stoichiometry PLD-deposited film, (ii) a CsBr-rich target is not an effective strategy to compensate Br-losses in the deposit and (iii) a Br-enriched target can be responsible for more effective intra-plume scattering through increased density of Br-species in the plasma plume. On the other hand, the observed Pb-enriched PLD-grown deposit also suggests that a PbBr_2_-rich target would favor further both Br-losses and Pb-enrichment.

Results discussed on the basis of the PLD mechanisms have demonstrated a systematic impact of the mass distribution in the plasma plume and disclosed experimental working conditions (in terms of fluence and preparation of a solvent-free target) making the PLD technique a successful approach to prepare CsPbBr_3_ films. To the best of our knowledge, the influence of the mass ratios among the involved elements (Cs, Pb and Br) on the composition of the PLD-deposited films has been investigated in no early literature report.

An interesting perspective of our findings and discussion is that proper changes in the PLD configuration could be a strategy to improve the collection of the Br species before their spreading beyond the side-part of the expanding plume and reducing Pb-excess in the deposit. This point, not included in this study, is being investigated and will be the subject of an upcoming paper.

## Figures and Tables

**Table 1:** Preparation parameters of the mechanochemically synthesized targets and PLD-deposited films under consideration in this study according to the nomenclature defined in the Experimental section. The composition of the targets was estimated by EDS. The composition of Film (300 °C) and Film(550 °C) was calculated by EDS. The composition of Film (2 J/cm^2^, 1:1) and Film (5 J/cm^2^, 1:1) was measured by RBS.

Sample	CsBr:PbBr_2_	F (J/cm^2^)	Cs_x_Pb_y_Br_z_	Film Thickness
Target (RT)	CsBr-rich	2	Cs_0.28_Pb_0.11_Br_0.61_	-
Target (300 °C)	CsBr-rich	2	Cs_0.21_Pb_0.16_Br_0.63_	-
Target (400 °C)	CsBr-rich	2	Cs_0.24_Pb_0.16_Br_0.60_	-
Target (500 °C)	CsBr-rich	2	Cs_0.24_Pb_0.17_Br_0.59_	-
Target (550 °C)	CsBr-rich	2	Cs_0.23_Pb_0.17_Br_0.60_	-
Film (300 °C)	CsBr-rich	2	Cs_0.21_Pb_0.24_Br_0.55_	390 nm
Film (550 °C)	CsBr-rich	2	Cs_0.21_Pb_0.21_Br_0.58_	365 nm
Film (2 J/cm^2^,1:1)	1:1	2	Cs_0.20_Pb_0.23_Br_0.57_	530 nm
Film (5 J/cm^2^,1:1)	1:1	5	Cs_0.22_Pb_0.23_Br_0.55_	
